# The Effects of Auditory Feedback Gait Training Using Smart Insole on Stroke Patients

**DOI:** 10.3390/brainsci11111377

**Published:** 2021-10-21

**Authors:** Junghyun Kim, Sangwoo Jung, Changho Song

**Affiliations:** 1 Department of Physical Therapy, Sahmyook University, Seoul 01795, Korea; kiking0@naver.com; 2 Department of Sports Rehabilitation, Gimcheon University, Gimcheon 39528, Korea; tkddn95@hanmail.net

**Keywords:** stroke, foot, gait, insole, weight-bearing

## Abstract

This study aimed to assess the effect of the auditory feedback gait training (AFGT) using smart insole on the gait variables, dynamic balance, and activities of daily living (ADL) of stroke patients. In this case, 45 chronic stroke patients who were diagnosed with a stroke before 6 months and could walk more than 10 m were included in this study. Participants were randomly allocated to the smart insole training group (*n* = 23), in which the AFGT system was used, or to the general gait training group (GGTG) (*n* = 22). Both groups completed conventional rehabilitation, including conventional physiotherapy and gait training, lasting 60 min per session, five times per week for 4 weeks. Instead of gait training, the smart insole training group received smart insole training twice per week for 4 weeks. Participants were assessed using the GAITRite for gait variables and Timed Up and Go test (TUG), Berg Balance Scale (BBS) for dynamic balance, and Modified Barthel Index (MBI) for ADL. The spatiotemporal gait parameters, symmetry of gait, TUG, BBS, and MBI in the smart insole training group were significantly improved compared to those in the GGTG (*p* < 0.05). The AFGT system approach is a helpful method for improving gait variables, dynamic balance, and ADL in chronic stroke patients.

## 1. Introduction

Individuals with post-stroke hemiparesis face difficulties in daily life that they have never experienced before [1]. Movements that were once easy, feel akin to a difficult task for a majority of stroke patients [2]. Physical disability is affected by reduced muscle activity, abnormal muscle tone, decreased coordination, and an influence of balance and gait [3,4].

When generally describing hemiparetic gait, it is asymmetric. The phenomenon where the heel of the affected side does not touch the ground in stance phase may be one of the main reasons for asymmetric gait [5]. Not being able to put the heel down on the ground may be seen in the whole stance phase or in the initial contact, depending on the patient. During this moment, the affected side may depend on the forefoot to perform the stance phase, and the unaffected side length of the stance phase shortens [6]. A decrease in stance phase leads to a decrease in the step length. Such impact shows the difference in gait phase between the unaffected side and the affected side. The spatial and temporal gait asymmetry can be observed even with the naked eye.

Adamczyk and Kuo [7] reported the contribution of foot length on gait efficiency and emphasized the clinical significance of the displacement of the anterior-posterior center of pressure in the foot. Chisholm et al. [8] stated that asymmetry of the anterior-posterior center of pressure between the lower extremities of stroke patients is significantly related to sensory-motor impairment. With the absence of heel-strike in stroke patients, the foot length, length of the foot contacting the floor during stance decreases, reducing the displacement of the anterior-posterior center of pressure. Consequently, this obstructs the rolling of the rocker system and reduces weight-bearing, thereby increasing the gait asymmetry.

We can estimate a stroke patient’s kinetic differences in their kinematic result value. The difference in step length may leads to the disparity in weight bearing for both feet. Decrease muscle activity of the paretic leg makes it difficult to shift weight to the affected side [9], as well as weight bearing towards the paretic leg during gait. Then, the single-limb support of the paretic side would decrease in a gait cycle due to the “learned nonuse”. 

Constraint-induced movement therapy (CIMT) is a suggested intervention for learned nonuse of the paretic limb. The concept is to force the use of the affected side, by limiting the movement of the unaffected side [10]. In the case of hemiparesis patients, they tend to not use the affected side. This may even lead to hemi-neglect, which made CIMT the most promising approach. CIMT was applied mainly on the upper limb [11]. Yet, in the lower limb, the role for locomotion is the main movement, so applying CIMT would not be easy. However, if the CIMT method for upper limb is modified to fit the lower limb, the concept of CIMT can be applied. Limiting the movement of the non-paretic leg and stimulating the movement of the paretic leg may limit the gait re-education [12].

The use of a shoe insole may be an effective tool to apply CIMT on the lower limb. The subject performs locomotion while standing on two feet, and through organic movement of the lower extremity, he can achieve an optimal gait pattern [13]. This enables for an effective rehabilitation of the lower extremity while also allowing free polyarthric movements and the use of spatiotemporal index from the footpad [14]. Attaching a micro-electronic device, with a small sensor on the insole, would enable the monitoring of daily life for a longer period of time [15,16]. It is now possible to track the changes of pattern and strategy over time by using the analysis for rehabilitation and training.

In stroke rehabilitation, it is possible to measure the velocity, stride length, cadence using the insole [17]. However, research with the insole was focused on measuring and assessment of gait, and not on the therapeutic approach [13,16,18]. A recent study introduced that the use of textured insole can contribute to gait symmetry of stroke patients. In the study the textured insole was placed on the unaffected side, and the modulation of gait parameters were observed. The negative feedback from the unaffected side decreased weight dependency, and increased stance phase and single support phase in the paretic leg. Thus, the study showed improvement of gait asymmetry using tactile feedback through insole [19].

The use of feedback is an effective approach for stroke patients to overcome the decrease in the use of paretic limb during gait [20]. Ma and his colleagues revealed the tactile feedback can be used for the gait symmetry [19]. We also observed the auditory feedback can be effective to achieve gait symmetry for stroke patients [21]. Our previous study applied auditory feedback with pneumatic-pressure insole to chronic stroke survivors. Through this study, we proved that gait asymmetry dramatically improved when 100% of the whole-body weight bears the insole under the affected side [22].

Therefore, we postulated that the use of auditory feedback with the pneumatic-pressure insole during gait training would lead to the control of weight dependency concentrated on the non-paretic. This would also increase the weight bearing time of the paretic leg during the stance phase. The aim of this study was to examine the effects on gait ability, dynamic balance and activities of daily living (ADL) using the Pneumatic pressure insole with auditory feedback in gait training (AFGT) for individuals with hemiparesis.

## 2. Materials and Methods

### 2.1. Subjects

The subjects were selected among chronic stroke inpatients of a medical rehabilitation center in Seoul. Inclusion criteria were chronic hemiplegic stroke patient six months past stroke diagnosis, a person with sufficient cognitive ability for exercise performance (more than 24 points in mono-mental state examination), and a person with the same or higher than Brunstromm recovery stage 3. Exclusion criteria were a person with neurological deficit other than the hemiplegic area; a person with an orthopedic problem such as lower limb fracture and peripheral nerve injury; a person with a visual problem, visual deficit, or hearing problem; and a person with more than double stroke history. Participants who had less than 80% of participation during the intervention were also excluded.

Before beginning the actual experiment, all subjects were informed of the research purpose and procedure, and only those who willingly signed the research consent form were allowed to participate. This study went through Sahmyook University Institutional review board. To decide sample size, the computer program G-power 3.19 was used, the significant level was set to 0.05, and statistical power was set to 0.8. The effect size was based on the variable of the affected single-limb support time through the pilot test, which is the critical factor of this study. The effect size was calculated to 0.90. The group sample size for the experiment was set to 21, considering a 10% dropout rate of 23.

### 2.2. Experimental Procedure

**Figure 1:**
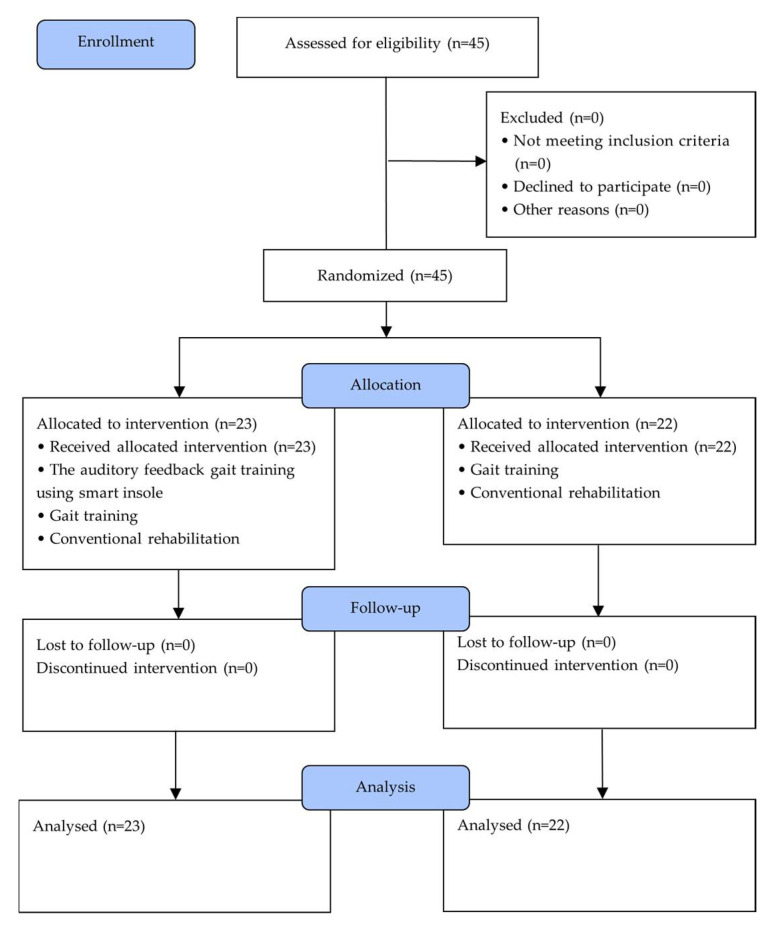
Experimental diagram.

Among chronic stroke inpatients at a medical center in Seoul, 45 subjects who fit the inclusion criteria were selected. After the pretest, factors such as gender and reason for the affected side were homogenized, and subjects were randomly allocated into two groups. Randomization software (Random allocation software, version 1.0, Isfahan University, Iran, 2004) minimized selection bias, and 23 subjects were put in AFGTG and 22 subjects in GGTG. AFGTG performed auditory feedback gait training using smart insole for 60 min per session, two sessions per week, for four consecutive weeks. Both groups performed gait training for 30 min each session, five times a week, for four weeks. Three examiners and three sub-examiners assessed the subject’s general information, motor recovery, lower limb muscle power, gait ability, postural balance, and activities in daily living through pretest (Figure 1). Subjects were not aware of the group to which they were assigned. During the study, patients who could no longer participate in the intervention program due to change of medical state, who had less than 80% participation rate were excluded. The patients and the assessing therapist were blinded to the group allocation [21].

### 2.3. Experimeantal Method

#### 2.3.1. Insole Auditory Feedback

We invented an insole typed auditory feedback device for practical weight-bearing training on the affected side during gait for stroke patients. Based on the pressure signal coming from both feet, the system analyzed the weight-bearing of both feet and set the aimed weight-bearing threshold. After the threshold is set, the device was made to provide auditory feedback when the weight-bearing reaches the aimed level.

**Figure 2:**
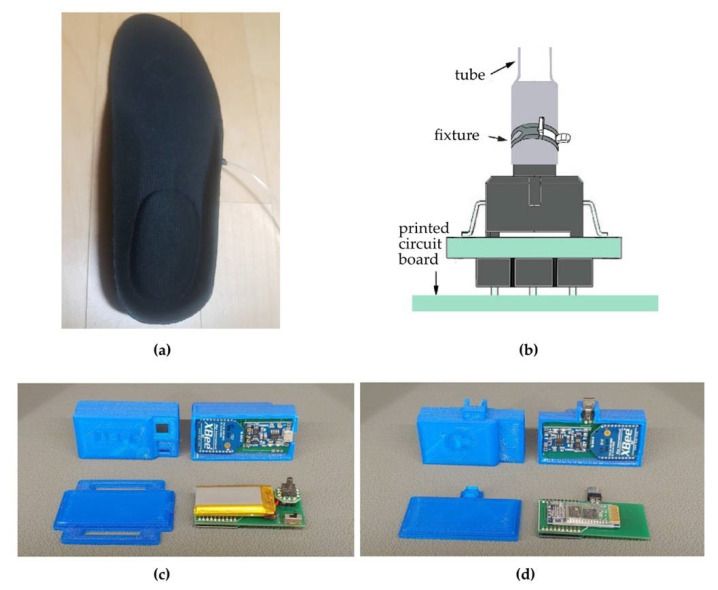
The custom-made air insole: (**a**) Air cap insole; (**b**) Pneumatic-pressor sensor. (**c**) Peripheral unit; (**d**) central unit.

The system consists of an air insole, peripheral unit, central unit, and mobile application. The air insole is custom-made (Figure 2), where it functions to send the pressure caused by the air cap inserted in the heel to the peripheral unit. The peripheral unit consists of two, one left and one right, and the pneumatic sensor (XGZP6847D, CFSensor Inc., Wuhu, China, 2017) measures the air pressure. The measured value was sent to the central unit through wireless ZigBee protocol (XBee 1mW Trace Antenna, 250 kbps data rate, SparkFun Electronics, Niwot, CO, USA). The central unit transmits the data from the two peripheral units to the mobile device’s application through the wireless Bluetooth protocol (HC-06, 2.1 Mbps data rate, HC-IT, Shenzhen, China).

The mobile application was developed using MIT App Inventor2. The pressure value generated in the air cap of each foot was normalized based on the sum of the weight-bearing pressures of the subject’s feet, and the weight-bearing value of each foot was measured. The threshold was set to provide auditory feedback when the affected side reaches appropriate weight support. The subjects were asked to perform the following two steps measurement procedure before the training session.

The first step was a normalization phase measuring the sum of the weight-bearing pressures of the heels from both feet. Subjects were asked to wear an insole-type auditory feedback device on both feet and stand in an anatomical position facing the front. The average value of the pressure from both feet was collected for 10 s. Once the total value is stored, all subsequent incoming pressure values were displayed as a percentage in proportion to the total weight-bearing pressures.

The second step was to set the aimed value for weight-bearing on the affected side. The subjects were asked to walk 3 m with comfortable speed. While walking, the therapist measures the maximum pressure generated by the patient’s heel. Based on the maximum pressure value, the therapist sets a threshold (%) to provide auditory feedback. Then the real-time pressure distribution value (%) from the affected limb was compared with the threshold. Auditory feedback was provided if the real-time pressure distribution exceeds the threshold.

The volume of auditory feedback increases when the real-time pressure value goes higher than the threshold. The subjects were instructed to make a louder sound as it is a positive signal of an achievement. During the gait training, therapists encouraged the subjects to keep motivated by making the sound louder and longer, inducing powerful and more extended weight bearing on the affected heel. Two phases of measurement were performed before every training session to adjust the subjects’ auditory feedback level.

#### 2.3.2. Auditory Feedback Gait Training Using the Smart Insole (AFGT)

These are characteristics of AFGT. It is customized to maximize effects on each subject by setting the aimed weight-bearing value following the subject’s ability. It was carried out at intervals of about three days. During the training session, the subjects had general gait training sessions applying the sense they had during the AFGT. The smart insole was applied in the patient’s footwear to reduce the sense of difference.

AFGT training consists of a warm-up, main exercise, and cool-down. In the warm-up phase, the subject performed 10 min of gait training at their speed. During the warm-up, the physiotherapist checked spasticity, contracture, and pain. The main exercise consists of ‘start from the standing posture’, ‘walk straight’, ‘walk the curved path’, ‘turn back’, and ‘stop’. Depending on the patient’s condition, the physiotherapist relevantly set up the program. The intervention was 10 min per set, in a total of 3 sets. In between sets, a 3–5 min break was given. The physiotherapist kept monitoring the subject and encouraged him/her to make sound continuously. During the main exercise, if the subject does not meet the goal or reach the goal too easily, he/she was measured again. Any patients complaining of dizziness or strenuousness during the training could take a break right away and return to training when feeling better. They were informed that they could stop out of their will any time. AFGT was carried out twice a week, 60 min each session, and more than three days apart between sessions to apply the sense they have learned in the general gait training.

#### 2.3.3. Conventional Rehabilitation Training

Conventional rehabilitation training consisted of physiotherapy and occupational therapy. The physiotherapy session consisted of neurodevelopmental therapy (NDT), balance training, and functional electrical stimulation (FES) on the upper limb. In an occupational therapy session, upper limb function improvement training was provided following their upper limb ability, and swallowing training was provided if the subject has dysphagia. We excluded any activities which can affect variables in our research during a conventional rehabilitation session. It was conducted for 60 min per day and five days a week.

#### 2.3.4. General Gait Training (GGT)

GGT is training to walk the gait track configured in the treatment room together with the therapist. The exercise consists of ‘start from the standing posture’, ‘walk straight’, ‘walk the curved path’, ‘turn back’, and ‘stop’. Depending on the patient’s condition, the physiotherapist relevantly set up the program. The intervention was 10 min per set, in a total of 2 sets. In between sets, a 3–5 min break was given. The physiotherapist kept monitoring the subject and encouraged him/her to make sound continuously. It was conducted for 30 min per day and five days a week.

### 2.4. Assessment Tools and Data Collection

#### 2.4.1. Gait Variables

Through a 3.66 × 0.61 m electronic gait mat, the GAITRite system (GAITRite, CIR Systems Inc., Franklin, NJ, USA, 2008) was used to collect and analyze spatiotemporal gait parameters gait velocity, cadence, stance time, swing time, stride length, and step length. As the participant walked over the gait mat, the machine measured the weight under the feet at a sampling rate of 80 Hz and transmitted the information to the computer through a serial interface cable. The device’s validity showed an excellent level of agreement with intraclass correlation coefficients between 0.92 and 0.99 [23]. In addition, the inter-rater reliability for step length, step, and stance time was also excellent (Intraclass correlation coefficient ≥ 0.94; lower limit confidence intervals (95% confidence interval ≥ 0.86) [24].

The spatiotemporal gait parameters were processed and analyzed using GAITRite GOLD, Version 3.2b software (CIR System Inc., Franklin, New jersey, USA, 2007). The orthoses used by subjects requiring these were not removed during walks. Subjects were asked to walk at their self-selected speed on an approximately 2-m walkway after the ‘que’ signal. The spatial gait parameters included step length, stride length, and gait line, while the temporal gait parameters were gait velocity, cadence, stride time, single-limb support time, single-step time, and double-limb support.

#### 2.4.2. Gait Symmetry

To quantify the extent of the temporal and spatial asymmetry of gait patterns, each was calculated as follows:

(1) $$\mathrm{Gait}\;\mathrm{symmetry}\;\mathrm{on}\;\mathrm{single}\;\mathrm{stance}\;\mathrm{time}=1-\frac{\mathrm{Single}\;\mathrm{support}\;\mathrm{time}\;\left(\mathrm{affected}\right)}{\mathrm{Single}\;\mathrm{support}\;\mathrm{time}\;\left(\mathrm{unaffected}\right)}$$

(2) $$\mathrm{Gait}\;\mathrm{symmetry}\;\mathrm{on}\;\mathrm{step}\;\mathrm{time}=1-\frac{\mathrm{Single}\;\mathrm{Step}\;\mathrm{time}\;\left(\mathrm{affected}\right)}{\mathrm{Single}\;\mathrm{Step}\;\mathrm{time}\;\left(\mathrm{unaffected}\right)}$$

(3) $$\mathrm{Gait}\;\mathrm{symmetry}\;\mathrm{on}\;\mathrm{single}\;\mathrm{stance}\;\mathrm{time}=1-\frac{\mathrm{Step}\;\mathrm{length}\;\left(\mathrm{affected}\right)}{\mathrm{Step}\;\mathrm{length}\;\left(\mathrm{unaffected}\right)}$$

In these ratios, “affected” referred to the affected lower extremity, and “unaffected” referred to the unaffected lower extremity. If the ratio is large, it indicates increased asymmetry [25].

#### 2.4.3. Dynamic Balance

1. Timed up and Go (TUG)

The timed “Up and Go” measures, in seconds, the time taken by an individual to stand up from a standard armchair, walk a distance of 3 m, turn, walk back to the chair, and sit down again. Inter- (0.99) and intra-rater (0.98) reliabilities are high [26].

2. Berg Balance Scale (BBS)

The BBS is a 14-item scale that quantitatively assesses balance and risk for falls. The items are scored from 0 to 4, with a score of 0 representing an inability to complete the task and 4 representing independent item completion. A global score is calculated out of 56 possible points. It is composed of 14 items that require subjects to maintain positions of varying difficulty and perform specific tasks such as standing and sitting unsupported, transfers (sit to stand and stand to sit), turn to look over shoulders, pick up an object from the floor, turn 360° and place alternate feet on a stool. The relative intra-rater reliability of the Berg Balance Scale was high, with a pooled estimate of 0.98 (95% CI 0.97 to 0.99). Relative inter-rater reliability was also high, with a pooled estimate of 0.97 (95% CI 0.96 to 0.98) [27].

#### 2.4.4. Modified Barthel Index (MBI)

The MBI measures the performance of ADLs and is a reliable and valid assessment for measuring functional status in stroke patients [28]. Each item, such as personal hygiene, bathing, feeding, toilet, stair climbing, dressing, bowel control, bladder control, ambulation, and wheelchair mobility and transfers, is scored numerically based on the extent of assistance the person requires. The maximum numerical score is 100. Scores of 0–24 denote total dependency, 25–49 denote severe dependency, 50–74 denote moderate dependency, and 75–90 denote little dependency. Inter- (0.99) and intra-rater (0.99) reliabilities are high [29].

#### 2.4.5. Mini Mental State Examination-Korea Version (MMSE-K)

The MMSE-K is a test designed to measure various cognitive functions and has proven reliability and validity in detecting severe or moderately advanced dementia. MMSE-K includes orientation, registration, attention, calculation, recall, language, and copying. It can be performed relatively easily and quickly and requires no additional equipment.

### 2.5. Data Analysis

Descriptive statistics were used to summarize the baseline characteristics data. Data analyses were performed using the Statistical Program for the Social Sciences ver. 20 for Windows (IBM; Armonk, New York, NY, USA). After confirming the normal distribution of the data using the Shapiro-Wilk test, we used the repeated measures ANOVA. The independent *t*-test was used to compare the gait parameters, TUG, BBS, and MBI between the AFGTG and GGTG. Comparisons between pre- and post-training data within each group were analyzed using the paired *t*-test. The effect size was computed using the formula effect size = d/SD where d is the mean difference scores, and SD is the standard deviation of the difference scores. The MDC is calculated by multiplying the standard error of measurement (SEM) by 1.96 to correspond to the 95% confidence interval and the square root of 2 to adjust for sampling from 2 different measurements [30]. The SEM is estimated as the pooled standard deviation (SD) of pre-and post-training assessments multiplied by the square root of (1 − r), where r is the intraclass correlation coefficient (ICC) [31]. Statistical significance was set at α < 0.05.

## 3. Results

In this case, 45 subjects participated in this study, all of whom completed the intervention and assessments. No differences were noted in the general characteristics of the two groups, including age, height, weight, body mass index, duration of a stroke, K-MMSE, paretic side, and stroke type (Table 1).

### 3.1. Gait

#### 3.1.1. Temporal Gait Parameter

After four weeks of training, the gait velocity significantly increased in both the AFGTG and GGTG (*p* < 0.05). Cadence, affected sidestep time, affected side single-limb support time, and double-limb support significantly differed in the AFGTG compared to the pre-measured value (*p* < 0.05). The GGTG showed no significant differences in any of these variables after training. The velocity, affected side step time, affected side single-limb support time, and double-limb support was significantly different between the two groups (*p* < 0.05) (Table 2).

#### 3.1.2. Spatial Gait Parameter

After training, the stride length, unaffected sidestep length and affected side gait line significantly differed in the AFGTG compared to the pre-measured value (*p* < 0.05). These also significantly differed between the AFGTG and GGTG (*p* < 0.05). The GGTG showed no significant differences in any of the variables after the intervention. The stride length, unaffected sidestep length and affected side gait line were significantly different between the AFGTG and GGTG (*p* < 0.05) (Table 3).

#### 3.1.3. Symmetry of Gait

The gait symmetries of the step length, step time, and single stance time were significantly different in the AFGTG compared to the pre-intervention findings (*p* < 0.05) and the GGTG (*p* < 0.05). The GGTG displayed no significant differences in any of these variables following the intervention (Table 4).

### 3.2. Dynamic Balance

Both the AFGTG and GGTG showed significant improvements in the TUG and BBS after training, with significantly better improvements in the AFGTG than in the GGTG (*p* < 0.05) (Table 5).

### 3.3. Activities of Daily Living (ADL)

Both the AFGTG and GGTG showed significant improvements in the MBI after training, with significantly better improvements in the AFGTG than in the GGTG (*p* < 0.05) (Table 5).

## 4. Discussion

This study aimed to investigate the short-term efficacy of the AFGT system in improving the gait variables, dynamic balance, and ADL in chronic stroke patients.

After four weeks of training, various gait parameters were assessed in the AFGTG and GGTG. Among the gait parameters that showed a change, gait velocity significantly changed in both groups, with the AFGTG seeing significant improvement compared to the GGTG. The gait velocity was significantly improved with a medium effect size of 0.77. This increase in gait velocity is positively correlated with the Brunnstrom stage, indicating symptom improvement [32]. However, other gait parameters should be checked because the increase in gait velocity is affected by the gait ability and compensatory strategies of various patterns [33]. In addition, it is difficult to explain the changes in the spatial gait parameters using only the gait velocity [34]. The GGTG did not show any significant gait parameters other than gait velocity; therefore, no change in gait ability or compensatory effect was observed. However, in the AFGTG, changes were confirmed in many gait parameters. The gait parameters with significant changes were divided into temporal and spatial gait parameters to determine the changes in the affected and unaffected sides.

First, among the AFGTG’s temporal gait parameters on the affected side, the single-limb support time increased significantly. In the affected side single-limb support time of the AFGTG, the difference value within the group improved to MDC% 61.43% beyond the threshold (MDC 0.03). MDC values are used to define a change in score that is not attributed to chance or measurement error. Therefore, the intra-group difference value of the affected side single-limb support time in the AFGTG was different due to training beyond the difference value of chance or measurement error. The AFGT insole used in this study produced louder auditory feedback depending on how much the set auditory feedback threshold has been exceeded. Therefore, it induced stronger and more continuous weight-bearing during gait. As the affected side single-limb support time improved, the affected side single-limb support time ratio and the unaffected side single-limb support time were also affected. Gait symmetry on single stance time showed a medium effect size of 0.78 between groups.

Second, the double-limb support was significantly reduced among the AFGTG’s temporal gait parameters on the unaffected side. The affected sidestep time was affected by the unaffected side single-limb support time and double-limb support. In our study, the affected sidestep time was significantly reduced (effect size 0.68) because the double-limb support decreased. In the double-limb support of the AFGTG, the difference value within the group improved to MDC% 42.14% beyond the threshold (MDC 2.28). Double-limb support is a stage in which the weight is transferred from one leg to the other, and the leg supporting the ground needs to be stable enough to the weight bear [35]. In the results of this study, the effect size of double-limb support was 1.24, indicating the large effect size. Based on these results, AFGT significantly influences the affected leg stability and weight transfer. No significant change in the unaffected side’s single-limb support time was noted. However, despite the decrease in the double-limb support, the unaffected sidestep time did not show a significant decrease due to the increase in the single-limb support time on the affected side. As a result, since the affected sidestep time decreased and the unaffected side step time did not change, the gait symmetry on step time was significantly improved with a medium effect size of 0.79.

Third, some significant changes were also observed in the spatial gait parameters of the affected side in the AFGTG. Using auditory feedback from the AFGT system, we induced heel contact during gait in stroke patients. As a result, our system showed a significant increase in the affected side’s gait line, the length of one foot (initial contact-to-push-off movement) touching the ground during gait. The affected side gait line in stroke patients has a short length because the affected side heel cannot touch the ground [6]. In the results of this study, training effectively improved the heel contact due to the lengthening of the affected side gait line after training. In the affected side gait line of the AFGTG, the difference value within the group improved to MDC% 34.87% beyond the threshold (MDC 0.69). The affected side gait line was significantly improved with a medium effect size of 0.67. It is also expected to affect the stance phase or hemiparetic gait pattern of the affected side. A previous study compared foot contact patterns during gait in 65 functionally ambulatory hemiplegic stroke patients and 30 healthy subjects and found that a higher Brunnstrom stage was associated with a longer gait line [32]. Physicians widely use the Brunnstrom stage to evaluate the movement function of stroke patients during rehabilitation [36].

An increase in the gait line indicates an improvement in the ability of the contact leg to maintain stability until the other foot touches the ground [37]. It also suggests a change in plantar flexor activity. Plantar flexor activity is also closely related to propulsion [38], negatively correlated with step length asymmetry [3]. Similarly, in our study, the unaffected sidestep length and gait symmetry on step length improved. In the unaffected sidestep length of the AFGTG, the difference value within the group improved to MDC% 52.75% beyond the threshold (MDC 2.03). The unaffected sidestep length was significantly improved with a large effect size of 0.97. This means that the stability of the affected leg was greatly improved as in the double-limb support. Additionally, there was an improvement in stride length (effect size 0.74), which is also believed to increase in the unaffected sidestep length.

As confirmed through the gait parameters, the stability and the gait velocity of the affected side increased. The TUG, used to evaluate gait ability, is not a simple gait velocity test as it involved turning back, sitting on a chair, and standing [39]. In this study, both the AFGTG and GGTG significantly decreased based on the pre-test and post-test results. In addition, the AFGTG showed a significant decrease in the TUG compared to the GGTG. The TUG improved with a medium effect size of 0.74. Among the factors influencing the TUG, fear of falling had a more significant influence than gait velocity [40]. However, since the improvement of the TUG alone cannot determine whether the fear of falls or gait ability has improved, other evaluation tools should also be checked.

Clinical assessments of human balance are evaluated based on different aspects of postural control. Postural control is the act of maintaining, achieving or restoring the line of gravity within the base of support [40]. While standing on both feet without the aid of an orthosis, the base of support relies on the feet and the smart insole inserts under the feet. The sensors used in the smart insole are capacitive, resistive piezoelectric, and piezoresistive sensors, which were selected based on their pressure range and sensitivity [41]. Smart insoles using these sensors can measure changes in pressure within the base of support due to postural control while standing. Therefore, in studies using the smart insole, the center of pressure is mainly measured based on the ground reaction force to evaluate postural control [19,42,43,44]. A pneumatic pressure-sensitive sensor was used in this study, but it was based on the ground reaction force measurement. Weight-bearing was trained by inducing heel contact on the affected side for 4 weeks. During this process, the patients had to perform postural control only with the affected leg. As a result, the overall balance of the AFGTG, confirmed through the BBS, significantly improved compared to that of the GGTG. The BBS improved with a medium effect size of 0.71.

As for the MBI score, measured to confirm the performance of ADLs, both the AFGTG and GGTG showed a significant difference in the post-test compared to the pre-test. In addition, the AFGTG showed a significant improvement compared to the GGTG. The MBI improved with a medium effect size of 0.59. As the AFGTG showed better improvement than the GGTG in the gait parameters, TUG, BBS, and MBI, the AFGT system was an effective gait training device.

Most smart insoles on the market use capacitive [45] or resistive sensors [46]. These sensors must use many sensors to observe the weight transfer in the smart insole [47]. The sensor’s small size limits the cell area that can be measured [48] and requires expert relocation whenever the user changes [49]. In addition, although the thin-film sensor is flexible, it is difficult to use for a long time due to continuous contact and friction caused by direct contact [50]. The ground reaction force that occurs on the ground is a three-dimensional force. but capacitive or resistive sensors can only measure vertical loads [48]. In contrast with capacitive and resistive sensors, a three-axis force sensor is more suitable for directly measuring three-dimensional ground reaction forces [51]. A product on the market called XSENS ForceShoe (XSENS North America Inc., Culver City, CA, USA) is based on a 6-axis force sensor for accurate ground reaction force measurement. However, since the sole height of the shoe is 3.2 cm and the total weight is 1.1 kg, this shoe interferes with normal gait [52]. There are three major disadvantages of products on the market. First, multiple sensors must be rearranged for each user. Second, the lifespan of the sensor is reduced due to the friction of direct contact. Third, the three-dimensional ground reaction force measurement is limited. Due to these shortcomings, the existing smart insoles were not suitable for gait training of stroke patients in terms of hardware. Since most smart insoles are designed for healthy adults, the software also supports feedback for healthy adults.

The smart insole used in this study was manufactured for gait training of stroke patients. Since it uses only one sensor, there is no need to relocate and it is placed on the heel, so it can fit anyone. An air cap was used as an intermediate medium for converting multidirectional forces into one direction. Due to the air cap because of direct contact and measure pressure in multiple directions. In addition, the volume and weight of the product are similar to smart insoles that use capacitive or resistive sensors, so it does not interfere with the wearer’s walking. The software was also developed as an app to provide feedback on the weight shift problem occurring while walking in stroke patients.

The AFGT system used in this study is a device that allows stroke patients to perform gait training anytime and anywhere when linked to a mobile device. Anyone can easily operate the application with only 10 min of training, and gait training tailored to a patient’s personalized target is possible. Therefore, this training system may be suitable for use in the home environment after discharge from the hospital. In the comparison between the AFGTG and GGTG, variables with significant differences showed a meaningful effect size between 0.59 and 1.24. This indicates that AFGTG is more effective than GGTG for gait training in stroke patients. This system was able to effect changes through the induction of heel contact on the affected side.

There were several limitations in this study. First, it was not able to control the stimulus for proprioception caused by the air cap. Secondly, the therapist could not control the difference in information transmission because he/she simultaneously looked at the mobile device and gave the subjects cues to walk. Finally, long-term effects were not confirmed. Therefore, it is essential to control proprioception, information transfer, and long-term effects in future studies.

## 5. Conclusions

The AFGT system can effectively improve gait variables, dynamic balance, and ADL by inducing heel contact in chronic stroke patients in patients with chronic stroke. In addition, based on a wireless communication protocol, it allows for gait training that does not interfere with the gait of stroke patients.

## Figures and Tables

**Table 1:** General Characteristics of Subject (*n* = 45).

	AFGTG (*n* = 23)	GGTG (*n* = 22)	χ^2^/*t*	*p*
Age (year)	61.78 ± 8.06	64.23 ± 0.57	0.875	0.386
Height (cm)	164.65 ± 7.49	162.45 ± 9.13	0.885	0.381
Weight (kg)	61.12 ± 7.61	61.47 ± 8.95	0.142	0.888
BMI (point)	22.51 ± 2.15	23.2 ± 2.00	1.111	0.273
Duration of stroke (month)	14.57 ± 7.24	16.86 ± 7.19	1.068	0.291
MMSE-K	25.78 ± 1.31	25.68 ± 0.99	0.289	0.774
Gender (male/female)	14/9	13/9	0.015	0.903
Paretic side (right/left)	12/11	16/6	2.021	0.155
Stroke type (infarction/hemorrhage)	16/7	13/9	0.538	0.463

Note. BMI = body mass index; MMSE-K = mini mental state examination-Korean.

**Table 2:** Changes of temporal gait parameter.

		AFGTG (*n* = 23)	GGTG (*n* = 22)	Time *F(p)*	Interaction *F(p)*	MDC MDC%
Velocity (cm/s)	Pre	29.65 ± 15.18	30.72 ± 14.20			
	Post	34.62 ± 16.88	32.63 ± 16.04	33.987	6.739	2.58
	Pre-Post	4.98 ± 4.47 ^†^	1.91 ± 3.35 ^†^	(0.000)	(0.013)	51.91
Cadence (step/min)	Pre	55.46 ± 13.15	56.21 ± 12.75			
	Post	59.98 ± 17.94	58.52 ± 16.61	10.546	1.113	
	Pre-Post	4.52 ± 7.96 ^†^	2.30 ± 5.94	(0.000)	(0.297)	
Stride time (sec)	Pre	2.36 ± 0.99	2.33 ± 1.01			
	Post	2.26 ± 1.05	2.29 ± 1.04	4.044	0.021	
	Pre-Post	−0.10 ± 0.28	−0.04 ± 0.17	(0.051)	(0.344)	
Affected side Step time (sec)	Pre	1.36 ± 0.50	1.35 ± 0.50			
	Post	1.23 ± 0.52	1.33 ± 0.51	12.976	5.188	0.09
	Pre-Post	−0.12 ± 0.16 ^†^	−0.03 ± 0.12	(0.001)	(0.028)	73.62
Unaffected side Step time (sec)	Pre	1.01 ± 0.50	0.98 ± 0.52			
	Post	1.03 ± 0.53	0.97 ± 0.55	0.092	0.511	
	Pre-Post	0.02 ± 0.16	−0.01 ± 0.12	(0.763)	(0.479)	
Unaffected side Sing limb support time (sec)	Pre	0.53 ± 0.14	0.57 ± 0.15			
	Post	0.54 ± 0.14	0.57 ± 0.16	0.163	0.002	
	Pre-Post	0.00 ± 0.05	0.00 ± 0.07	(0.668)	(0.961)	
Affected side Sing limb support time (sec)	Pre	0.39 ± 0.14	0.40 ± 0.14			
	Post	0.44 ± 0.14	0.40 ± 0.14	3.377	7.021	0.03
	Pre-Post	0.05 ± 0.05 ^†^	0.00 ± 0.07	(0.073)	(0.011)	61.43
Double limb support (%)	Pre	57.70 ± 14.39	55.29 ± 13.73			
	Post	52.28 ± 15.86	54.15 ± 12.79	40.633	17.361	2.28
	Pre-Post	−5.41 ± 3.95 ^†^	−1.13 ± 2.82	(0.000)	(0.000)	42.14

Values are expressed as mean ± standard deviation. ^†^ means significant difference between group.

**Table 3:** Changes of spatial gait parameter.

		AFGTG (*n* = 23)	GGTG (*n* = 22)	Time *F(p)*	Interaction *F(p)*	MDC MDC%
Stride length (cm)	Pre	62.26 ± 21.97	64.25 ± 20.77			
	Post	67.31 ± 18.66	65.11 ± 18.84	12.337	6.179	3.76
	Pre-Post	5.05 ± 6.50 ^†^	0.86 ± 4.59	(0.001)	(0.017)	74.35
Affected side Step length (cm)	Pre	33.27 ± 12.74	34.08 ± 12.11			
	Post	34.48 ± 10.44	34.13 ± 10.29	1.138	0.978	
	Pre-Post	1.21 ± 3.94	0.05 ± 3.93	(0.292)	(0.328)	
Unaffected side Step length (cm)	Pre	28.99 ± 9.56	30.17 ± 9.02			
	Post	32.83 ± 8.76	30.99 ± 9.02	24.960	10.510	2.03
	Pre-Post	3.84 ± 3.51 ^†^	0.82 ± 2.68	(0.000)	(0.002)	52.75
Affected side gait line (cm)	Pre	22.71 ± 3.8	22.4 ± 3.59			
	Post	24.69 ± 4.47	22.61 ± 2.99	7.678	5.028	0.69
	Pre-Post	1.98 ± 1.20 ^†^	0.21 ± 3.59	(0.008)	(0.030)	34.87

Values are expressed as mean ± standard deviation. ^†^ means significant difference between group.

**Table 4:** Changes in gait symmetry.

		AFGTG (*n* = 23)	GGTG (*n* = 22)	Time *F(p)*	Interaction *F(p)*	MDC MDC%
Gait symmetry on step length (score)	Pre	0.19 ± 0.10	0.17 ± 0.11			
	Post	0.12 ± 0.08	0.20 ± 0.19	1.090	4.218	0.06
	Pre-Post	−0.07 ± 0.10 ^†^	0.02 ± 0.17	(0.302)	(0.046)	91.66
Gait symmetry on step time (score)	Pre	0.40 ± 0.23	0.46 ± 0.29			
	Post	0.23 ± 0.11	0.49 ± 0.43	3.377	7.021	0.12
	Pre-Post	−0.16 ± 0.20 ^†^	0.03 ± 0.28	(0.073)	(0.011)	71.83
Gait symmetry on single stance time (score)	Pre	0.39 ± 0.14	0.40 ± 0.14			
	Post	0.44 ± 0.14	0.40 ± 0.14	7.025	6.853	0.03
	Pre-Post	0.05 ± 0.05 ^†^	0.00 ± 0.07	(0.011)	(0.012)	61.43

Values are expressed as mean ± standard deviation. ^†^ means significant difference between group.

**Table 5:** Changes in dynamic balance and ADL results.

		AFGTG (*n* = 23)	GGTG (*n* = 22)	Time *F(p)*	Interaction *F(p)*	MDC MDC%
TUG (sec)	Pre	31.76 ± 7.08	32.53 ± 6.74			
	Post	27.59 ± 5.24	30.81 ± 6.54	35.633	6.152	1.76
	Pre-Post	−4.16 ± 3.05 ^†^	−1.72 ± 3.55 ^†^	(0.000)	(0.017)	42.37
BBS (point)	Pre	29.42 ± 9.39	29.48 ± 8.95			
	Post	33.48 ± 8.55	31.28 ± 8.30	37.997	5.632	1.61
	Pre-Post	4.07 ±2.78 ^†^	1.81 ± 3.58 ^†^	(0.000)	(0.022)	39.47
MBI (score)	Pre	48.68 ± 11.40	51.14 ± 12.26			
	Post	55.85 ± 14.18	52.79 ± 12.54	52.187	20.415	2.79
	Pre-Post	7.18 ± 4.83 ^†^	1.65 ± 12.54 ^†^	(0.000)	(0.000)	38.92

Values are expressed as mean ± standard deviation. ^†^ means significant difference between group.
